# Intrafloral Color Modularity in a Bee-Pollinated Orchid

**DOI:** 10.3389/fpls.2020.589300

**Published:** 2020-11-09

**Authors:** João Marcelo Robazzi Bignelli Valente Aguiar, Artur Antunes Maciel, Pamela Cristina Santana, Francismeire Jane Telles, Pedro Joaquim Bergamo, Paulo Eugênio Oliveira, Vinicius Lourenço Garcia Brito

**Affiliations:** ^1^Programa de Pós-Graduação em Entomologia, Departamento de Biologia, Faculdade de Filosofia, Ciências e Letras de Ribeirão Preto, Universidade de São Paulo, Ribeirão Preto, Brazil; ^2^Programa de Pós-Graduação em Ecologia e Conservação dos Recursos Naturais, Instituto de Biologia, Universidade Federal de Uberlândia, Uberlândia, Brazil; ^3^Programa de Pós-Graduação em Ecologia, Departamento de Ecologia, Universidade de São Paulo, São Paulo, Brazil; ^4^Jardim Botânico do Rio de Janeiro, Rio de Janeiro, Brazil; ^5^Instituto de Biologia, Universidade Federal de Uberlândia, Uberlândia, Brazil

**Keywords:** bees, color signaling, color properties, integration, Orchidaceae

## Abstract

Flower color has been studied in different ecological levels of organization, from individuals to communities. However, it is unclear how color is structured at the intrafloral level. In bee-pollinated flowers, the unidirectional gradient in color purity and pollen mimicry are two common processes to explain intrafloral color patterns. Considering that floral traits are often integrated, usually reflecting evolutionary modules under pollinator-mediated selection, we hypothesize that such intrafloral color patterns are structured by intrafloral color modules as perceived by bee color vision system. Here, we studied the tropical bee-pollinated orchid *Cattleya walkeriana*, given its intrafloral color complexity and variation among individuals. Considering bee color vision, we investigated if intrafloral color modules arose among intrafloral patches (tip or base of the sepals, petals, and labellum). We expected a separate color module between the labellum patches (the main attractive structure in orchids) and petals and sepals. We measured the color reflectance and calculated the photoreceptor excitation, spectral purity, hue, and the chromatic contrast of the floral structures in the hexagon color model. Spectral purity (saturation) was higher in the labellum tip in comparison to petals and sepals, generating a unidirectional gradient. Labellum base presented a less saturated yellow UV-absorbing color, which may reflect a pollen mimicry strategy. *C. walkeriana* presented three intrafloral color modules corresponding to the color of petals and sepals, the color of the labellum tip, and the color of labellum base. These color modules were unrelated to the development of floral structures. Given the importance of intrafloral color patterns in bee attraction and guidance, our results suggest that intrafloral patterns could be the outcome of evolutionary color modularization under pollinator-mediated selection.

## Introduction

Floral color is one of the most important signals in plant-pollinator communication, usually increasing pollinators efficiency in detecting flowers in nature and improving their efficacy in obtaining floral resources and pollinate the flower (Wester and Lunau, 2017). Through the visual communication between pollinators and plants, selective pressures can be imposed, generating a coevolutionary process. In that process, plants will adapt to the perception of pollinators, and pollinators will adapt to floral signals. These processes can generate floral color patterns structured on different ecological organization levels, from communities (Shrestha et al., 2014; Bergamo et al., 2018; Camargo et al., 2019), populations (Mascó et al., 2004; Newman et al., 2012), to the inter-individual level within a population, i.e., color polymorphisms (Bergamo et al., 2016; Jiménez-López et al., 2019; Aguiar et al., 2020). Regardless of the organization level, most studies describe color properties such as petal spectral purity and hue as a single value per flower (Rohde et al., 2013; Shrestha et al., 2013; Koethe et al., 2016). However, a single flower may display distinct colors, creating intrafloral color patterns that attract and guide pollinators (Lunau et al., 1996). Generally, intrafloral color variation in angiosperms is a result from differential pigment accumulation regulated by tissue-specific transcription during the process of pigment biosynthesis (Martins et al., 2017). Despite understudied, intrafloral color patterns are especially common in bee-pollinated flowers (Camargo et al., 2019), and are important features favoring legitimate visits (Medel et al., 2003; Leonard and Papaj, 2011; Papiorek et al., 2016).

There are two non-exclusive general hypotheses to explain the evolution of intrafloral color patterns in bee-pollinated flowers, both related to the selective pressures imposed by pollinator perception and behavior. The first is the saturation gradient, which corresponds to a centripetal increase in spectral purity from flower periphery to the center. Bees orientate themselves toward the center of the flower following a gradient of centripetally increasing color saturation produced by floral guides (Lunau, 1990). When the spectral purity (saturation) of the corolla is lower than the spectral purity of the guides, bumblebees and honeybees innately react to guides, approaching and inspecting them (Lunau, 1990; Lunau et al., 2009). Several structures are examples of intrafloral guides such as spots (Thomas et al., 2009), bull’s eye patterns (Papiorek et al., 2016) and pigmentation over the petal vasculature or veins, often referred to as resource guides (Whitney et al., 2013). The second hypothesis to explain intrafloral color patterns is related to the “pollen mimicry” strategy, based on the fact that many flowering plants display pollen- and stamen-like structures, enhancing or replacing the visual signals associated with pollen itself (Heuschen et al., 2005; Lunau et al., 2017). The strategy consists in presenting yellow structures or areas of similar color to those of anthers and pollen. Bees react to yellow UV-absorbing areas in natural or artificial flowers and prefer those over flowers presenting no such signals (Papiorek et al., 2016; Telles et al., 2020). The presence of these yellow signals can potentially increase plant fitness, by inducing visits (Lunau, 2006; Duffy and Johnson, 2015; Lunau et al., 2017).

Intrafloral color patterns are probably the outcome of a complex interplay among different selective pressures on flower pigments, including those exerted by the preferences of pollinators (Schemske and Bradshaw, 1999; Medel et al., 2003; Caruso, 2004; Papiorek et al., 2016; Caruso et al., 2019), abiotic factors, and genetic and developmental constraints (Ashman and Majetic, 2006; Klingenberg, 2014; Bergamo et al., 2018). As pollinators can act as an important source of natural selection, Raissa Berg proposed that correlation among reproductive traits would generate floral integration, as a consequence of the selective pressures on traits that promoted successfully pollination (Berg, 1960; Alcantara et al., 2013; Armbruster et al., 2014; Fornoni et al., 2016). However, selection could act differently on distinct floral structures, due to different functions, generating distinct trait modules within flowers (“intrafloral modularity”) (Armbruster et al., 1999, 2014; Pérez et al., 2007; Ordano et al., 2008; Baranzelli et al., 2014; Dellinger et al., 2019). Intrafloral modularity is the tendency of some floral traits to covary among themselves while being independent of other traits (Armbruster and Wege, 2019). Moreover, pollination efficacy is thought to increase as a result of intrafloral modularity (Armbruster and Wege, 2019). The same mechanisms that generates intrafloral modularity could be applied to the comprehension of intrafloral color patterns.

Considering the interaction with pollinators, different color areas within a flower could attract pollinators first from a distance, and then guide their approach at a close range (Lunau, 1993). In doing so, complex intrafloral signals could be decoupled in different modules that maximize plant fitness through consistent pollinator orientation. Taking into consideration the two intrafloral color variation hypotheses explained above, pollinator-mediated integration (and further modularization in the different floral structures) could be the mechanism behind the centripetal increase in color saturation. Integration in color hue could also lead to yellow UV-absorbing modules, leading to a pollen mimicry pattern.

In orchids, floral color often shows intraspecific polymorphism and many orchids present nectar guides and differences in color between floral parts (Darwin, 1877; Aguiar et al., 2012; Sletvold et al., 2016; Pansarin et al., 2018). Furthermore, orchid flowers present a specialized structure, the labellum, a modified petal which is usually distinct from petals and sepals regarding both color and morphology (Darwin, 1877; van der Pijl and Dodson, 1966; Fay and Chase, 2009). In this case, while petals and sepals attract the pollinators from long distances, the labellum is related to pollinator attraction and behavior in short distances (van der Pijl and Dodson, 1966; Fay and Chase, 2009). Given their potential different functions, sepals have evolved semi-independently from the petals, and the labellum has evolved semi-independently from petals (Mondragón-Palomino and Theißen, 2009). In addition, there was a phylogenetic conservatism during labellum evolution in comparison to the high lability of other petals and sepals in a specific orchid group (*Cirrhopetalum* alliance), revealing that pollinator-mediated selection could have a role in the modular evolution of orchid flowers (Hu et al., 2019), which could also influence intrafloral color patterns. Also, the hybrid orchid *Cattleya* “KOVA,” presents a spatiotemporal variation in pigments accumulation during floral development, regulated by three different transcription factors (Li et al., 2020). As a result, a low accumulation of pigments in sepals and lateral petals promote a pale pink color, while a high accumulation of cyanine pigments in the labellum tip and of carotenoids in the labellum base promote purple-red and yellow colorations, respectively (Li et al., 2020). Therefore, considering different ecological functions and the differences in pigment accumulation in floral tissues, it would be expected that the perceived intrafloral pattern in *Cattleya* sp. is a result of intra-floral color modularity. Although intraspecific color variation is well documented in orchids (Gigord et al., 2002; Juillet and Scopece, 2010; Ackerman et al., 2011; Kagawa and Takimoto, 2016; Aguiar and Pansarin, 2019; Aguiar et al., 2020), intrafloral color variation has received little attention.

In this study, we investigated the intrafloral color patterns of the tropical bee-pollinated orchid *Cattleya walkeriana* Gardner focusing on interindividual variation of hue and spectral purity taking into account the bee-color vision system. Considering the differential morphology of floral structures and their known functions, we expected to find an increasing pattern of saturation toward the flower center, with sepals and petals less saturated than the labellum. We also describe the presence of a yellow UV-absorbing center, which could work as floral guide to bees during approach, in a pollen mimicry mechanism. We also expected to find less color variation among plants in the center of their flowers when compared to their peripheries. Finally, we expected to find three intrafloral color modules corresponding to the color of petals and sepals, the color of the labellum tip and the color of labellum base.

## Materials and Methods

### Plant Species and Growth Conditions

**FIGURE 1:**
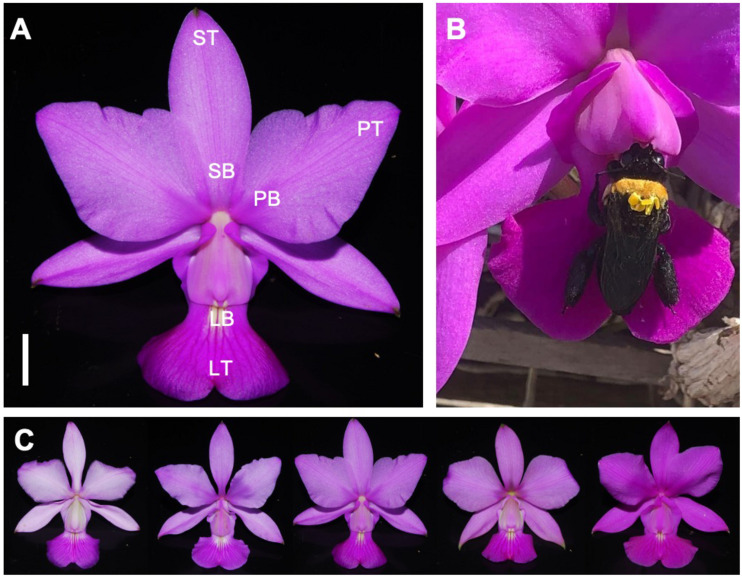
Flowers of *Cattleya walkeriana.* **(A)** intrafloral color patches sampled; **(B)** a visit of a bee pollinator carrying some pollinia; **(C)** color variation among sampled individuals. PB, petal base; PT, petal tip; SB, sepal base; ST, sepal tip; LB, labellum base; LT, labellum tip. Bar = 1 cm. Photos **(A,C)**: Vinícius L. G. Brito; photo **(B)**: courtesy of Joaquim Barreto Carneiro Filho.

*Cattleya walkeriana* Gardner (Orchidaceae: Laeliinae) is a widespread bee-pollinated orchid (Silva and Milaneze-Gutierre, 2005) found on rocks or trees in areas near lakes, rivers, and swamps in different regions of Cerrado, the Brazilian savanna (Faria et al., 2002). Flowers present a color pattern with pinkish to dark pink sepals and petals, and a dark pink labellum with a yellow area on its base (Figure 1). Some species of the same genus have been repeatedly reported to be self-compatible, pollinator-dependent and nectarless, deceiving bees and hummingbirds during the pollination process (Smidt et al., 2006; Caballero-Villalobos et al., 2017). Despite some records of *C. walkeriana* flowers visited by bees (Figure 1B), detailed information about the reproduction of this species is still unknown. This orchid bloom mostly from May to July and the duration of flower anthesis is relatively long (from 7 to 10 days). Breeding systems tests revealed that *C. walkeriana* is self-compatible but depends on pollinators to set fruits (Supplementary Table S1). Spontaneous selfing and apomixis treatments did not produce any fruit, whereas hand-selfing and hand-crossing treatments resulted in 34% and 54% fruit-set, respectively (Supplementary Table S1).

All the plants used in this study were collected during the flowering period of 2011 in four natural areas of Goiás State, Brazil. Due to common predatory orchid collection practice, sampling areas are available only under request. Plants were collected at least 10 m apart to each other and were kept in the same growth conditions in the greenhouse of Orquidário Paranaíba, in Itumbiara municipality, Goiás State, Brazil. Plants were cultivated in clay pots (20 cm in diameter and 15 cm in depth) using tree barks as substrate. To reduce the possible effects of environment on color differences (Schemske and Bierzychudek, 2001; Dafni et al., 2020), individuals were cultivated in the same natural light and temperature conditions and were watered at least twice a day.

### Reflectance Measurements

To study floral color properties as perceived by bees, we measured the spectral reflectance of the tip and base of different floral structures, which we named as floral patches throughout this text, from 30 plant individuals (one flower per individual) during the flowering season of 2015. Floral structures were divided in sepals, petals, and labellum (Figure 1A). All the measurements were performed in recently open flowers, following the sequence from sepal to labellum in each plant, measuring it at the tip and the base of each floral structure (Figure 1), totaling 180 measurements. Spectral reflectance curves were collected using a portable spectrophotometer with a built-in light source (Jaz; Ocean Optics Inc., Dunedin, FL, United States; light source range from 189.91 nm until 896.99 nm) coupled with an optic fiber reflection probe (R400-7-UV-VIS Jaz; Ocean Optics Inc., Dunedin, FL, United States). To calibrate the spectrophotometer, we used a white diffuse reflectance pattern (WS-1, Ocean Optics Inc., Dunedin, FL, United States) and covered the spectrophotometer entrance as black standard. All the measurements were made at an angle of 90° with the probe positioned 2 mm from the sample.

### Color Modeling, Color Properties and Color Distances

We estimated the color locus occupied by each floral patch (tip and base of each floral structure) in the hexagon color space (Chittka, 1992). To that, we used the floral patch reflectance, a green leaf standard as the background (Chittka and Kevan, 2005), and a daylight illumination (D65; Wyszecki and Stiles, 1982), together with the spectral sensitivity of *Bombus terrestris* (Peitsch et al., 1992). From that, we estimated the excitation values of each the UV, blue and green photoreceptors and posteriorly the position (X and Y coordinate values) on the hexagon color space (Chittka, 1992). Once we build the model and estimated the color locus of each floral patch, we measured the color properties, which are hue (i.e., the pure state of color, derived from the different pigment composition in the flower, which leads to what we know as different colors such as red or blue) and the relative spectral purity (i.e., the hue intensity, related to different pigment concentration in the flower, originating dark and light versions of the same hue; Dormont et al., 2019). We also calculated the color contrast against the background (i.e., the color difference between an object and its background).

In the hexagon model, color hue can be measured as the angle formed between the color locus and any axis chosen arbitrarily (Chittka, 1992; Shrestha et al., 2014). Therefore, we measured color hue as the angle between the *x*-axis of the hexagon model and the line crossing the hexagon center and the color loci (Chittka, 1992). Spectral purity was obtained considering the perceptual distance between the locus of each floral patch measured and the background, divided by the distance between the corresponding spectral locus and the maximal spectral purity at the same locus (Lunau et al., 1996). Finally, color contrast against the background was measured as the distance between the color locus and the central point of the color hexagon, which represents the locus of the standard green background (Chittka, 1992).

To estimate color variation within patches among plants, we calculated the distance, in hexagon units, among the color loci belonging to the same floral patch. In the hexagon model, color distances may be estimated in two different ways that also have different biological meanings. Euclidian color distances between loci is a proxy for chromatic contrast, while the angular distance between loci corresponds to the difference in color hue. We are aware that bees behave differently to colors on different hexagons subsets, in a way that similar Euclidean distances might not elicit similar discrimination responses by bees (Dyer and Chittka, 2004; Telles and Rodríguez-Gironés, 2015), yet the bee hexagon color space model is a simple and reliable tool to access the kind of information we are aiming to evaluate in this study (Gawryszewski, 2017).

### Data Analyses

As spectral purity and contrast against background were highly correlated in our sample (r_Pearson_ = 0.94; df = 178; *p* < 0.001), we decided to perform the statistical analyses only using the spectral purity because it is directly related to the saturation gradient hypothesis. To analyze the intrafloral color patterns of *C. walkeriana*, we compared the spectral purity of each floral patch using a linear mixed-effect model. In this model, the spectral purity was considered as the response variable, while floral structures (sepal, petal, and labellum), the position of reflection curve measurement (tip and base) as well as the interaction among these factors were considered the explanatory variables. The identity of plant individuals was considered a random effect. Afterward, we ran *post hoc* pairwise multiple comparison *t* tests between all floral patches with false discovery rate (FDR) at a significance level of 0.05 (Benjamini and Hochberg, 1995). We also compared the values of color hue among floral patches using a circular one-way ANOVA. Unfortunately, the current tools to perform circular ANOVA do not allow interaction terms, and thus, we could not include an interaction between floral structures and the position of reflection curve measurement. Therefore, color hue was considered the response variable, and the color patches (sepal tip, sepal base, petal tip, petal base, labellum tip, and labellum base as levels) as the explanatory variables. We used the Watson’s two-sample test of homogeneity for *post hoc* comparisons. As such test does not allow any correction for multiple comparisons, we used a conservative significance level of 0.001.

We used both color distances, Euclidean and angular, to analyze color variation within patches between pairs of plants (totaling 435 comparison pairs). In the first case, we used a linear mixed model considering the squared root of Euclidian distances between loci of the same floral patch as the response variable and, floral structures (sepal, petal, and labellum), the measurement position (tip and base) and the interaction between them as explanatory variables. The plant pair were considered a random term. Afterward, we ran a pairwise multiple comparisons *t*-test with FDR procedures at significance level of 0.05 (Benjamini and Hochberg, 1995). We back transformed the values to the Euclidian distances when building the plot corresponding to this analysis for better visualization. The angular distances between the loci of pairs of patches were analyzed using a circular one-way ANOVA. The angular color distance between loci of the same floral patch was considered as the response variable, and the color patches as the explanatory variable. We used the Watson’s two-sample test of homogeneity for *post hoc* comparisons with a conservative significance level of 0.001. We validated all the previous models by visually checking whether the residuals were evenly distributed around the fitted values.

Finally, we used the covariance-ratio (CR) test (Adams, 2016) to explicitly test the hypothesis that intrafloral color patterns of *C. walkeriana* correspond to three intrafloral color modules, i.e., the color of petals and sepals, the color of the labellum tip and the color of labellum base. The CR uses pairwise covariances to quantify modular structures and ranges from zero to positive values. A low CR value (i.e., between zero and one) indicates relatively less covariation among modules than that found within modules, characterizing a more modular structure in the data. On the other hand, a higher CR value describe higher covariation among modules. In this analysis, we used the normalized UV, blue and green photoreceptor excitation values as variables rather than X and Y coordinates in the hexagon because photoreceptor excitation values have direct biological meaning related to color as perceived by bees. The significance of the CR value found was evaluated by 10000 permutations in which photoreceptor excitation values for each flower patch were randomly assigned to one of the three designated color modules. The proportion of permuted CR values lower than the original was treated as an estimate of the significance level of the test (Adams, 2016).

All analyses were performed in R (ver. 3.6.2; R Development Core Team, 2020), using the packages *pavo* (Maia et al., 2019), *circular* (Agostinelli and Lund, 2013), *lme4* (Bates et al., 2015), *lmerTest* (Kuznetsova et al., 2017), *geomorph* (Adams and Otárola-Castillo, 2013), and *ggplot2* (Wickham, 2016).

## Results

### Floral Color Properties

Reflectance of the tip and base of sepals and petals, as well as those of the tip of the labellum were similar, reflecting light in blue and red wavelengths, while labellum base reflected mostly in green and red wavelengths (Supplementary Figure S1). When considering the hexagon space, floral patches occupied different color sections (Supplementary Figure S2). The loci of sepals and petals were in the blue section of the hexagon, while labellum tip loci fell in the UV-blue section and labellum base loci were in blue-green section (Supplementary Figure S2).

**FIGURE 2:**
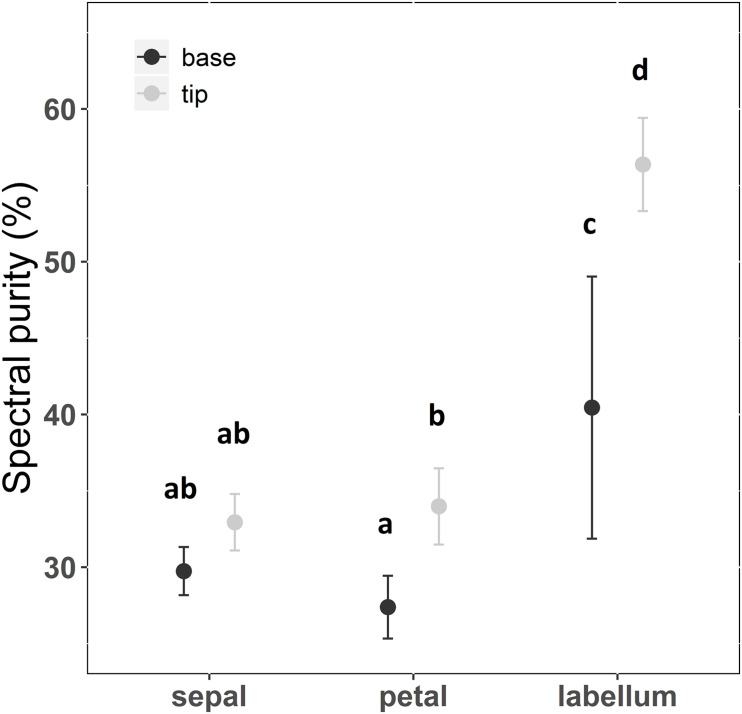
Spectral purity of floral patches of *Cattleya walkeriana*. Dots indicate the mean value and bars the 95% confidence interval. Different letters show statistical difference after pairwise multiple comparison among floral patches, with a false discovery rate at a significance level of 0.05. *N* = 30 individuals.

**FIGURE 3:**
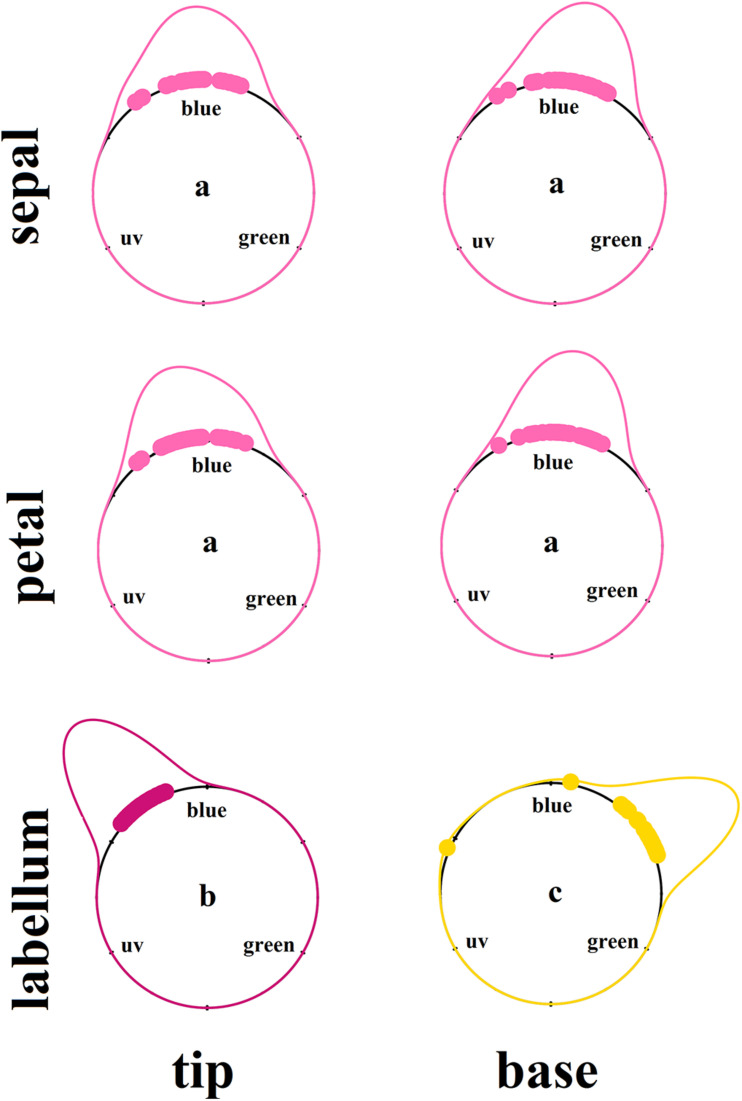
Kernel density estimation of the color hue of floral patches of *Cattleya walkeriana*. Dots indicate the raw hue values of each color loci. Density curves color represent the floral structure as perceived by the human eyes. Letters indicate statistical difference after Watson’s two-sample test of homogeneity, at significance level of 0.001. *N* = 30 individuals.

There was a significant interaction between floral structure (sepal, petal, or labellum) and position of reflection curve measurement (tip or base) (*F* = 5.728; df = 2; *p* < 0.01; Supplementary Table S2 and Figure 2). Sepals and petals presented the lowest purity irrespective of position (Supplementary Table S2). In general, the labellum presented spectral purity 56% higher than petals and sepals regardless the measurement position (Supplementary Table S2 and Figure 2). Thus, considering the different floral structure identity, spectral purity increased from flower periphery to center, with labellum presenting higher spectral purity than sepals and petals. However, contrary to our expectations, taking in consideration the variation within floral structure, petal base exhibited lower purity than petal tip, and labellum tip presented higher purity than labellum base (Supplementary Table S2 and Figure 2). Considering hue on the hexagon space, sepals and petals did not differ in this color property, while the labellum presented a distinct hue (Figure 3 and Supplementary Table S2). Moreover, hue differed between labellum tip and base (Figure 3 and Supplementary Table S2).

### Color Distances Among Plant Individuals

**FIGURE 4:**
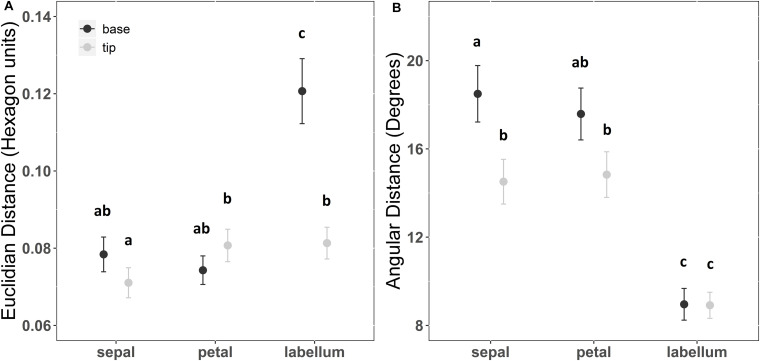
Chromatic contrasts **(A)** and hue differences **(B)** within floral patches among individuals of *Cattleya walkeriana*. Chromatic contrast was measured as the Euclidian distance while hue difference was measured as the angular distance among pairs of color loci in color hexagon. Dots indicate the mean value and bars the 95% confidence interval. Letters above chromatic contrast values indicate statistical differences after a pairwise multiple comparison among floral patches with a false discovery rate at a significance level of 0.05. Letters above angular distance values indicate statistical difference after Watson’s two-sample test of homogeneity with a significance level of 0.001.

The interaction between floral structure and position of reflection curve measurement explained color Euclidian distances among pairs of plant individuals (*F* = 30.681; df = 2; *p* < 0.001). In general, sepals, petals and labellum tips presented Euclidian distance among individuals from 0.07 to 0.09 hexagon units (Supplementary Table S3). However, Euclidian distance in labellum bases among individuals could reach 0.12 hexagon units. Therefore, Euclidian distance among pairs (i.e., color contrast) of plant individuals were 58% higher in labellum bases than in any other combination of floral structure and position (Figure 4A and Supplementary Table S3).

The angular color distance among plant individuals differed between floral structure and position of reflection curve measurement (*F* = 65.7; df = 5; *p* < 0.001). In general, angular color distance among individuals in sepals and petals varied from 14° to 19°, while angular color distance among individuals were approximately 9° for both labellum tip and base. The mean angular distance (i.e., color hue difference) of the labellum among plant individuals were lower than the mean angular distance of any other combination of floral structure and position (Figure 4B and Supplementary Table S3).

### Intrafloral Color Modularity

The CR value found for the three proposed color modules in *C. walkeriana* was 0.42 (confidence interval: 0.37–0.76; *p* < 0.001) indicating that intrafloral color patterns correspond to three independent color modules.

## Discussion

Many flowers present intrafloral color pattern, and such pattern has a role in attraction and guidance of pollinators to the flowers’ resource (Medel et al., 2003; Leonard and Papaj, 2011; Papiorek et al., 2016). Here we investigated the intrafloral color pattern as well as their variation among plants of *C. walkeriana*, a bee-pollinated orchid (Silva and Milaneze-Gutierre, 2005), which depends on pollinators to set their fruits (Supplementary Table S1). Therefore, intrafloral color patterns may play a role in *C. walkeriana* reproduction if such pattern affects bee attraction. As we expected, the flowers of this orchid presented a centripetally increasing spectral purity, being petals and sepals (periphery) less saturated than the labellum (center). Also, the yellow UV-absorbing hue of labellum base showed the lowest variation among floral patches. Several bee-pollinated flowers are yellow UV-absorbing, a color often used by bees to locate resources (Heuschen et al., 2005; Lunau et al., 2017). Finally, we found modularity in intrafloral color, being the colors of sepals and petals, labellum tip, and labellum base distinct modules as perceived by bees. We expected to find this color modularity as, in orchids, the labellum is often the main floral structure in terms of pollinator attraction and pollinator orientation, usually presenting different features compared to the other floral elements (van der Pijl and Dodson, 1966; Fay and Chase, 2009; Armbruster and Wege, 2019).

The great majority of the bee-pollinated *Cattleya* spp. studied so far are food deceptive (Smidt et al., 2006; Silva-Pereira et al., 2007; Caballero-Villalobos et al., 2017). If this is also the case of *C. walkeriana*, the intrafloral color variation pattern found here could be even more advantageous in this rewardlessness scenario. In this sense, the intrafloral color pattern generated by color modularity would improve generalized food deceptive exploitation of pollinators based on their innate cognitive preferences (Jersáková et al., 2006; Johnson and Schiestl, 2016).

### Centripetal Increase on Color Spectral Purity

Although petals and labellum presented higher spectral purity at their tip than at their base, there was a general increase of spectral purity in *C. walkeriana* from the periphery to the center of the flower, peaking at the labellum. This pattern could be a consequence of selective pressure imposed by pollinators, as it is linked to a higher attraction and a more effective guidance of pollinators to floral reproductive structures. Spectral purity has been demonstrated to play a role on bee attraction and guidance when approaching and landing on flowers (Rohde et al., 2013), likely favoring correct placement and pickup of pollen on the bee body. Moreover, orchid floral resources are usually located at the labellum in center of the flower (Fay and Chase, 2009). A centripetal increase of color spectral purity could enhance pollination service of effective pollinators in the studied orchid, even in the case when flowers offer no reward at all. Some pollinating bees have innate preference for colors with higher spectral purity (Lunau et al., 1996) and such preference could generate the selective pressures that modulate the observed intrafloral color pattern in *C. walkeriana*. Contrary to our expectations, the petals tip presented higher spectral purity than the base, with a small decrease from the periphery (34.0%) to the center (24.7%). However, as the spectral purity of the labellum is much higher (40.5–56.4%) than the petals, the small variation in spectral purity within sepals and petals should not influence bees’ attraction toward the labellum. Further behavioral tests would be useful to support this hypothesis. Within the labellum, the very center of the flower (labellum base) also showed lower spectral purity in relation to labellum tip (although this saturation was still higher in comparison with petals and sepals). A low saturation in labellum base when compared to tip was unexpected by our former hypothesis that intrafloral color saturation should increase centripetally (Lunau, 1990). However, contrasting with the difference in spectral purity at the petals, the variation found at the labellum may be explained by another color constraint in central floral elements: pollen mimicry in the labellum base.

### Pollen Mimicry

The labellum base, although presenting lower spectral purity in comparison with labellum tip, presented a completely different hue from the other floral parts. Moreover, color hue variation among individuals was exceptionally low within this floral patch when compared to sepals and petals. In other words, flowers of *C. walkeriana* showed a strong color hue constraint in their labellum base no matter their spectral purity. This is common in specialized bee-pollinated flowers, which usually present less diverse inner colors when compared to the peripheral colors (Heuschen et al., 2005). In *C. walkeriana*, the labellum base is yellow UV-absorbent, which is known to be a color category related to pollen mimicry in bee-pollinated flowers (Lunau, 2000). Many insects seek for pollen during flower visit, and the visual signals of pollen are well conserved across the flowering plants (Lunau, 1995; Lunau et al., 2017). Therefore, presenting yellow UV-absorbent areas or structures may be an attraction advantage to the plants (Johnson and Schiestl, 2016). Thus, although the very center of the flowers of *C. walkeriana* present less color saturation than its immediate surroundings, contrary to the expected centripetal increase of spectral purity, the presence of a yellow UV-absorbent spot, which is very conserved in its hue among individuals, could be a pollen mimicking strategy to increase floral attractiveness for bees regardless its lower saturation.

### Intrafloral Color Modularity

Sepals and petals formed a single intrafloral color module, while the labellum tip and labellum base varied independently. The morphology of orchid flowers is regulated by the expression of four different classes of DEF-like genes, even if they are part of the same or different floral whorls. While class 1 and 2 genes are activated in all floral structures, class 3 is activated in the petals and labellum, and class 4 only at the labellum. This promotes the striking intrafloral differentiation found in orchid flowers, and it is assumed to be the genetic base of the vast diversity found in the flowers of Orchidaceae (Mondragón-Palomino and Theißen, 2009). Therefore, the overall similarity in sepals and lateral petals found here could be explained by the common expression of organ identity genes shared between sepals and petals (DEF-like classes 1 and 2) but not in labellum. Also, evidence from the literature shows that the labellum is under differential pollinator-mediated selection in relation to sepals and petals (Mondragón-Palomino and Theißen, 2009; Hu et al., 2019). This was especially strong for the labellum base color. The strong constraint in color hue associated to the high variation in color saturation of the labellum base may have driven the evolution of this floral patch as a completely different intrafloral color module. Contrasting these results with the morphology of sepals, petals, and labellum in orchids, highlights that floral morphology could have evolved semi-independently from the intrafloral color patches, being constrained by different developmental routes of floral pigments and driven by different selective forces.

Patterns of integration and modularity in floral traits among individuals can be determined by different underlying biological processes, such as development, environment, functionality, and genetics (Klingenberg, 2014). In our study, environmental conditions were controlled, and flowers were analyzed in the same developmental stage (i.e., during the floral anthesis). Thus, the intrafloral color modularity found in *C. walkeriana* is not a consequence of environmental variation, yet it represents the evolutionary history experienced by this species. Given the role of color on bee attraction and guidance, color modularity in *C. walkeriana* might be, therefore, the product of selective pressures imposed by pollinators. Armbruster and Wege (2019) proposed that the evolution of both floral and intra-floral modularity is a consequence of the degree of the floral phenotypic specialization more than the ecological specialization. Orchids present flowers phenotypically highly specialized with bilateral symmetry and the pollinator needs to be highly oriented with the fertile parts to the pollinia be correctly deposited (Darwin, 1877; van der Pijl and Dodson, 1966). This floral phenotypical specialization highlights the role that pollinators had in orchids evolutionary history, including the intrafloral color modules, as shown here. Despite floral integration and modularity have been showed only for morphological floral traits such as the size of structures (Berg, 1960; Armbruster et al., 2014), here we propose that modularity could arise in other floral traits under pollinator-mediated selection, such as color.

## Conclusion

To our knowledge, our study is the first to show intrafloral color modularity considering the color vision system of the pollinator. This modularization may be the result of selective pressures imposed by the visual preferences of pollinators and the need of consistency in pollinators visits to be efficient. In *C. walkeriana*, both the centripetal increase on color saturation and the yellow UV-absorbent patch of the labellum base create three different intrafloral color modules that are not entirely related to the morphological modules typically found in orchids (Hu et al., 2019). Such modules can be defined as: (1) the less saturated and variable color of sepals and petals, (2) the color of the labellum tip, which showed the highest color saturation, probably as an outcome of the selective pressure imposed by the innate preference of bees for centripetally color saturation in flowers (Lunau, 1990; Rohde et al., 2013), and (3) the color of the labellum base, which shows low hue variation among individuals and is probably an outcome of the selective pressure derived from the innate preference of bees for pollen-like colors (Lunau, 2000; Heuschen et al., 2005). Thus, this work provides an example of how we should take into consideration pollinators’ perception and cognition to understand how they could impose selective pressures which shapes intrafloral color patterns evolution. Further studies should investigate floral integration and modularity across distinct traits in order to broaden our understanding of the evolution of intrafloral patterns.

## Data Availability Statement

The raw data supporting the conclusions of this article will be made available by the authors, without undue reservation.

## Author Contributions

JA and VB wrote the first draft of the manuscript. AM and VB designed the study and collected and analyzed the data. PS, PB, FT, and PO contributed significantly in the discussion of the data and reviewed and contributed to the text of the manuscript. All authors contributed to the article and approved the submitted version.

## Conflict of Interest

The authors declare that the research was conducted in the absence of any commercial or financial relationships that could be construed as a potential conflict of interest.
