# Supplementary material for: Intrafloral Color Modularity in a Bee-Pollinated Orchid
Source: Front Plant Sci. 2020 Nov 9;11:589300. doi: 10.3389/fpls.2020.589300 (PMC7693458; doi:10.3389/fpls.2020.589300)
Supplement: Supplementary file 1 [file Data_Sheet_1.docx]

# Supplementary Material

# Intrafloral color modularity in a bee-pollinated orchid

João Marcelo Robazzi Bignelli Valente Aguiar^1^

Artur Antunes Maciel^2^

Pamela Cristina Santana^3^

Francismeire Jane Telles^2^

Pedro Joaquim Bergamo^4^

Paulo Eugênio Oliveira^5^

Vinicius Lourenço Garcia Brito^5,^*

^1^ Programa de Pós-graduação em Entomologia, Departamento de Biologia, Faculdade de Filosofia, Ciências e Letras de Ribeirão Preto, Universidade de São Paulo. Ribeirão Preto, Brazil, 14040-900.

^2^ Programa de Pós-graduação em Ecologia e Conservação dos Recursos Naturais, Instituto de Biologia, Universidade Federal de Ubelândia. Uberlândia, Brazil, 38405-315.

^3^ Programa de Pós-graduação em Ecologia Departamento de Ecologia, Universidade de São Paulo; São Paulo, Brazil.

^4^Jardim Botânico do Rio de Janeiro, Rio de Janeiro, Brazil

^5^ Instituto de Biologia, Universidade Federal de Uberlândia. Uberlândia, Brazil, 38405-315.

*Author for correspondence. Email: viniciusduartina@gmail.com

**Breeding system**

Prior to color measurements, we tested the breeding system of 52 plant individuals of *C. walkeriana*. During the flowering period of 2012, and using previously bagged flowers we applied the following treatments: 1) apomixis: pollinia were removed from flowers; 2) spontaneous selfing: flowers remained bagged until wilt; 3) hand-selfing: pollinia were transferred to the stigmatic surface of the same flower and; 4) hand-crossing: pollinia were transferred to the stigmatic surface of a flower from a different individual. After experimental manipulations, all treated flowers were immediately bagged. Following senescence, we counted the fruit set of each treatment as an evidence of the reproductive success.

**Table S1.** Fruit set from flower treatments in *Catleya walkeriana*. In parenthesis: the number of fruits and treated flowers.

| **Flower Treatment** | **Fruit set %** |
| --- | --- |
| Apomixis | **0 (0 / 35)** |
| Spontaneous selfing | **0 (0 / 35)** |
| Hand-selfing | **34 (12 / 35)** |
| Hand-crossing | **54 (19 / 35)** |

**Table S2.** Mean spectral purity and color hue (± 95% CI) of floral patches of *Catleya walkeriana*. Color spectral purity was estimated as the proportion between the distance of the color locus from the achromatic centre of the hexagon and the distance of the corresponding spectral locus representing the maximal spectral purity considering bumblebees’ photoreceptor excitation from the same point. Color hue was measured as the angle between the x-axis of the hexagon model and the line crossing the hexagon centre and the color. Different letters following spectral purity values indicate statistical difference after pairwise multiple comparison among floral patches with false discovery rate with a significance level of 0.05. Letters following color hue values indicate statistical difference after Watson's two-sample test of homogeneity with a significance level of 0.001.

| **Floral Structure** | **Position of reflection curve measurement** | **N** | **Spetctral purity (%)** | **Color Hue (^O^)** |
| --- | --- | --- | --- | --- |
| Sepal | base | 30 | 29,7 ± 1,6 ^ab^ | 94.7 ± 0.3 ^a^ |
| Sepal | tip | 30 | 32,9 ± 1,8 ^ab^ | 82.1 ± 0.3 ^a^ |
| Petal | base | 30 | 27,4 ± 2,1 ^a^ | 98.1 ± 0.3 ^a^ |
| Petal | tip | 30 | 34,0 ± 2,5 ^b^ | 83.8 ± 0.2 ^a^ |
| Labellum | base | 30 | 40,5 ± 8,6 ^c^ | 33.3 ± 0.4 ^b^ |
| Labellum | tip | 30 | 56,4 ± 3,0 ^d^ | 126.6 ± 0.1 ^c^ |

**Table S3.** Mean chromatic contrast and hue difference (± 95% CI) in floral patches among individuals of *Catleya walkeriana*. Chromatic contrast among individuals was estimated as the Euclidian distance between pairs of color loci while the difference in color hue was estimated as the angular distance between loci. Different letters following chromatic contrast values indicate statistical difference after pairwise multiple comparison among floral patches with false discovery rate with a significance level of 0.05. Letters following angular distance values indicate statistical difference after Watson's two-sample test of homogeneity with a significance level of 0.001.

| **Floral Structure** | **Position of reflection curve measurement** | **n** | **Euclidian Distance (Hexagon units)** | **Angular Distance (^O^)** |
| --- | --- | --- | --- | --- |
| Sepal | base | 435 | 0.08 ± 0.005 ^ab^ | 18.50 ± 1.28 ^a^ |
| Sepal | tip | 435 | 0.07 ± 0.004 ^a^ | 14.51 ± 1.01 ^b^ |
| Petal | base | 435 | 0.07 ± 0.004 ^ab^ | 17.58 ± 1.17 ^ab^ |
| Petal | tip | 435 | 0.08 ± 0.004 ^b^ | 14.84 ± 1.04 ^b^ |
| Labellum | base | 435 | 0.12 ± 0.008 ^c^ | 8.96 ± 0.72 ^c^ |
| Labellum | tip | 435 | 0.08 ± 0.004 ^b^ | 8.91 ± 0.59 ^c^ |

**
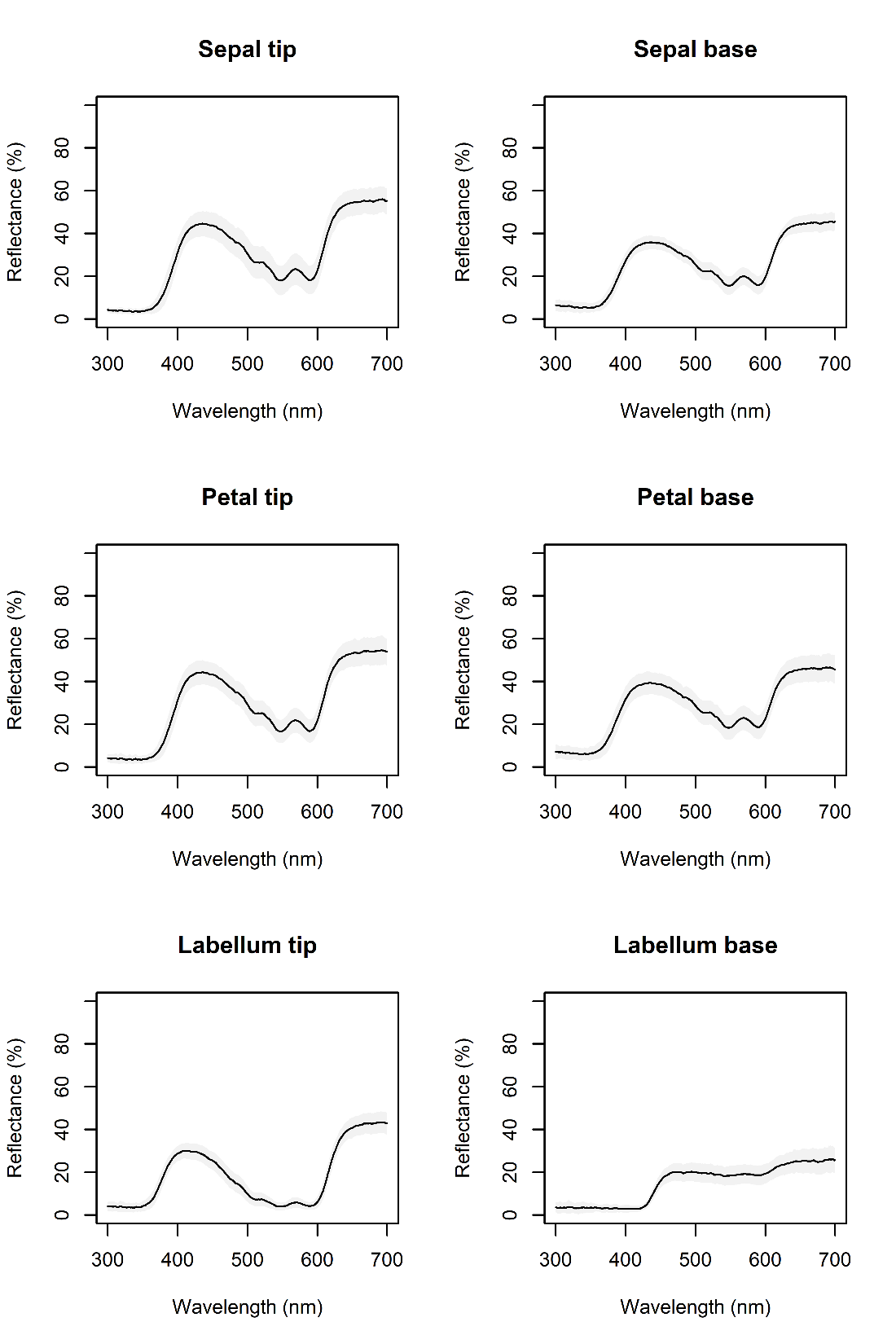
**

**Figure S1.** Average reflectance curves of floral patches of *Catleya walkeriana*. Shadows indicate the standard deviation. N = 30 individuals.

##
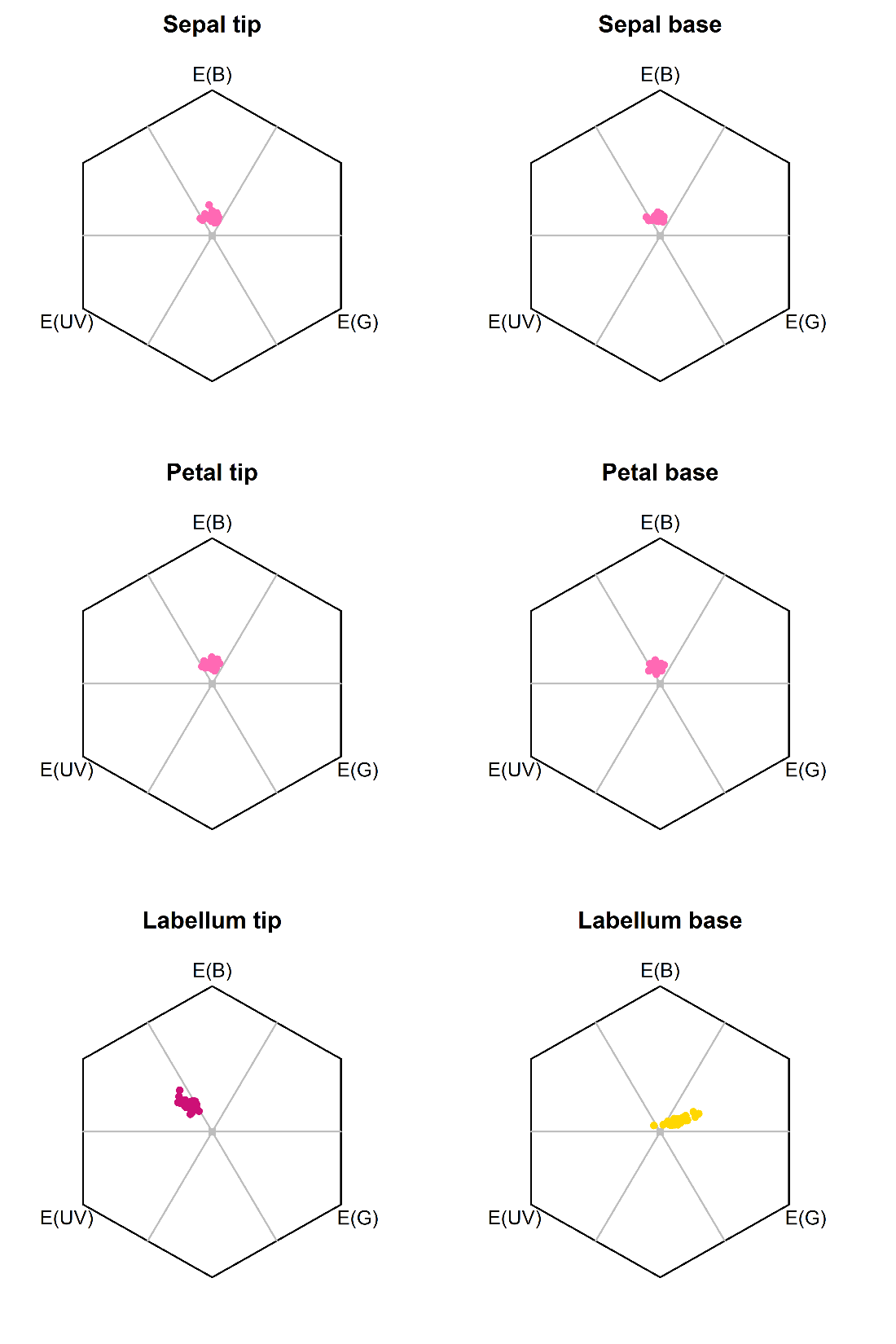


**Figure S2.** Color hexagon plots showing the coordinates of floral patches of *Catleya walkeriana.* In this model, the hexagon is defined by three photoreceptor excitation axes linked at an angle of 120^o^. To build these hexagons we used a green leaf standard background, a daylight illumination (D65) and the spectral sensitivity of each photoreceptor type of *Bombus terrestris*. Color loci are estimated by the relative excitation of each photoreceptor. Hexagon slices defined by grey lines represent the approximate color section within the hexagon. N = 30 individuals.
